# The Future of Material Scientists in an Age of Artificial Intelligence

**DOI:** 10.1002/advs.202401401

**Published:** 2024-03-13

**Authors:** Ayman Maqsood, Chen Chen, T. Jesper Jacobsson

**Affiliations:** ^1^ Institute of Photoelectronic Thin Film Devices and Technology Key Laboratory of Photoelectronic Thin Film Devices and Technology of Tianjin College of Electronic Information and Optical Engineering Nankai University Tianjin 300350 China; ^2^ Department of Physics Chemistry and Biology (IFM) Linköping University Linköping 581 83 Sweden

**Keywords:** artificial intelligence, closed‐loop experimentation, machine learning, material science

## Abstract

Material science has historically evolved in tandem with advancements in technologies for characterization, synthesis, and computation. Another type of technology to add to this mix is machine learning (ML) and artificial intelligence (AI). Now increasingly sophisticated AI‐models are seen that can solve progressively harder problems across a variety of fields. From a material science perspective, it is indisputable that machine learning and artificial intelligence offer a potent toolkit with the potential to substantially accelerate research efforts in areas such as the development and discovery of new functional materials. Less clear is how to best harness this development, what new skill sets will be required, and how it may affect established research practices. In this paper, those question are explored with respect to increasingly more sophisticated ML/AI‐approaches. To structure the discussion, a conceptual framework of an AI‐ladder is introduced. This AI‐ladder ranges from basic data‐fitting techniques to more advanced functionalities such as semi‐autonomous experimentation, experimental design, knowledge generation, hypothesis formulation, and the orchestration of specialized AI modules as stepping‐stones toward general artificial intelligence. This ladder metaphor provides a hierarchical framework for contemplating the opportunities, challenges, and evolving skill sets required to stay competitive in the age of artificial intelligence.

## Graphical abstract

In this perspective, the implications of adopting increasingly advanced AI technologies in materials science are discussed. It is considered how to best utilize AI technologies, identify the necessary new skills, and examine the impact they may have on traditional research methodologies. Central to the discussion is a conceptual AI ladder that spans from elementary data fitting to general artificial intelligence.

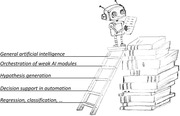

## 1 Introduction

Throughout human history, the discovery of new materials has transformed and reshaped societies. Given the needs we have and the vanishingly small part of the chemical space that has been explored, there is no reason to believe the future will be any different. New functional materials will be pivotal in enabling breakthroughs in applications ranging from renewable energy, clean air and water, space exploration, next‐generation nuclear power, energy storage, catalysis, and quantum computing. Advances in material science may also give us things like room‐temperature superconductors and other innovations still belonging to the realm of science fiction.

Unfortunately, discovering and developing new functional materials is an inherently complex challenge that often is slow and labor‐intensive. Theories that could guide rational material design are scarce, and there is no counterpart to the Schrödinger equation for predicting synthesisability. Each potential material is further associated with a nearly infinite parameter space of variables that can affect the synthesis, and once synthesized, the properties are influenced by a multitude of factors such as microstructure, imperfections, defects, and impurities. Moreover, feedback frequently depends on a range of highly specialized characterization techniques, each necessitating time, resources, and specialized training to operate effectively.

Great needs and infinite possibilities provide strong incentives for improving the rate of development. Historically, advancements in material science have been driven by human ingenuity, curiosity, and experimental expertise. This has been further augmented by increasingly advanced characterization techniques, more powerful computing capabilities, and an ever‐expanding body of knowledge. Recently, machine learning (ML) and artificial intelligence (AI) have emerged as vital components of this toolkit, showing great potential for playing an increasingly important role in accelerating the pace of materials discovery and development. This paper will discuss the potential impact that increasingly capable ML/AI systems may have in material science.

In some sense, ML parallels traditional statistics: often useful, sometimes misinterpreted, occasional pivotal for generating new insights, but generally only a small part of the scientific narrative. However, we are now witnessing a rapid evolution in artificial intelligence where increasingly capable systems are solving problems that were until recently considered to be the stuff of science fiction. AI systems have already surpassed human abilities in games such as chess,^[^ ^1^ ^]^ Jeopardy,^[^ ^2^ ^]^ and GO,^[^ ^3^ ^]^ can predict how proteins fold,^[^ ^4^ ^]^ and are even capable of autonomous driving.^[^ ^5^ ^]^ Furthermore, Large language models (LLM) like ChatGPT can produce text nearly indistinguishable from human‐generated content,^[^ ^6^ ^]^ and text‐to‐image systems based on latent diffusion models can create visually stunning art.^[^ ^7^ ^]^ What's more, we are beginning to see how different AI algorithms and subsystems are being integrated to tackle increasingly complex problems.^[^ ^8^ ^]^ This synergy among AI components has the potential to start a new industrial revolution,^[^ ^9^, ^10^ ^]^ but may also render a significant portion of existing jobs obsolete.^[^ ^11^, ^12^ ^]^

From the perspective of material science, this rapid development of artificial intelligence prompts a series of compelling questions. To what extent can AI accelerate the development of new materials? Does materials science present unique challenges for AI, or can generalized algorithms suffice? Could AI fundamentally revolutionize the way materials science is conducted? What new skill sets will be required, and which existing practices will need to evolve for that to happen? How much of the research process could potentially be delegated to AI entities? May there even be a conceivable future in which today's materials researchers and their skill sets becomes obsolete, replaced entirely by AI systems? Or is the perceived significance, importance, and future impact of AI greatly inflated and merely a contemporary hype?

**Figure 1:**
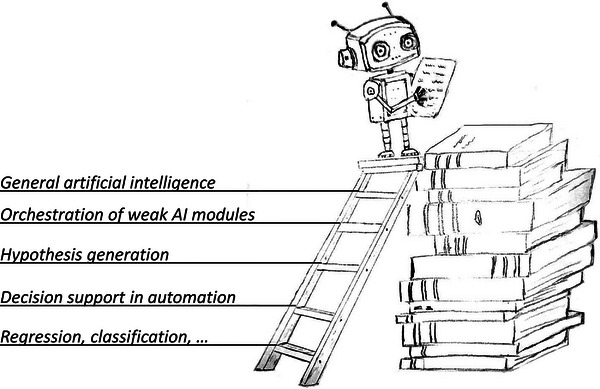
The increasing complexity and sophistication of AI systems can be thought about in terms of an AI‐ladder that is stretching from basic linear regression all the way up to general artificial intelligence and beyond. In this paper we are discussing AI in five broad categories illustrated in the ladder in the figure, but one can imagine an arbitrary number of rungs on this ladder.

In this perspective, we delve into those questions by examining current trends and making informed projections into the near future. AI is a broad concept, ranging from relatively simple algorithms to sophisticated universal function approximators^[^ ^13^ ^]^ that when integrated with robotics, can autonomously interact with the physical world. We can conceptualize the complexity of AI systems with a ladder (see **Figure** **1**) where each rung represents increasingly advanced capabilities – spanning from basic data fitting to semi‐autonomous experimentation, experimental design, knowledge creation, general artificial intelligence, and beyond. We frame our discussion on the use of AI in materials science around this ladder metaphor, which provide a hierarchical framework for contemplating the opportunities, challenges, and evolving skill sets that may be required. While the primary focus and examples in this paper relate to materials science, much of the analysis is likely applicable also to other scientific disciplines.

## 2 The First Rung Up the Ladder

### 2.1 ML‐Models and What to Do with Them

The initial rung of the ML/AI ladder resemble traditional statistics, albeit approached with a somewhat different mindset. At its most basic, this includes straightforward techniques like linear regression. More broadly, this stage often entails employing models trained on limited datasets to accomplish specific tasks, which are usually oriented toward regression or classification. The overarching aim is to create a statistical model that can serve as a surrogate for a physical model. This is especially useful when a physical model is either too intricate to derive or entirely elusive. In essence, this first step is about leveraging statistical inference to provide an alternative way of understanding and predicting outcomes in situations where traditional physical models may not be practical.

There is a plethora of machine learning models, with some of the more widely used ones being linear regression, decision trees, Extra Trees, Random Forest (RF), AdaBoost (ABoost), Gradient Boosting (GBoost), Extreme Gradient Boosting (XGBoost), Support Vector Machines (SVM), and Multi‐Layer Perceptrons (MLP), among others. Even for those with minimal experience in a programming language such as Python, utilizing these models have become increasingly accessible thanks to well‐maintained open‐source libraries such as Scikit‐learn,^[^ ^14^ ^]^ TensorFlow,^[^ ^15^ ^]^ Keras,^[^ ^16^ ^]^ PyTorch,^[^ ^17^ ^]^ etc. For those interested in delving into the mathematical underpinnings of these algorithms or learning how to implement them in code, there are numerous high‐quality resources available.^[^ ^18^, ^19^ ^]^ Here we will instead focus on the applications and implications of trained models.

Regression and classification models essentially serve as shortcuts, enabling a reasonable prediction of the outcomes of experiments, or the properties of materials, without synthesising the material and conducting the experiments. This becomes particularly useful when comprehensive physics‐based models are not available, but when data has been collected. One can then train models that establish relationships between for example material composition and solar cell efficiency,^[^ ^20^, ^21^, ^22^, ^23^ ^]^ or molecular structure to attributes such as solubility,^[^ ^24^ ^]^ toxicity,^[^ ^25^ ^]^ or antibacterial effect.^[^ ^26^ ^]^ Once trained, such a models can be used for virtual screening of new molecules and materials to identify promising candidates for further detailed investigation, thus dramatically reducing the number of experiments needed. A recent notable example is given by Stokes et al., who employed such an approach to discover a new type of antibiotics.^[^ ^27^ ^]^

A trained model can also serve as a tool for introspection, enabling deeper understanding of the data and relationships within it. With techniques such as associative rule mining,^[^ ^28^ ^]^ SHAP‐analysis (SHapley Additive exPlanations),^[^ ^29^ ^]^ correlation plots, and feature weighting it is possible to assess the significance of individual variables or clusters of variables. This knowledge can then guide the formulation of new heuristics and hypotheses for subsequent experiments, potentially paving the way for more robust physics‐based models and transferable insights. Utilizing machine learning models in this manner aligns well with the current academic publishing paradigm, wherein a high‐quality study typically introduces a new material, proposes a novel synthesis route, or offers insights into the material's behavior under specific conditions.

Another valuable application for machine learning models arises in scenarios where physics‐based models do exist but are computationally expensive, such as in the case of quantum mechanical simulations. For example, Density Functional Theory (DFT), which is the workhorse of molecular and materials simulations, is relatively affordable for limited‐scale screening but is still constrained by computational costs. By using existing DFT data, a neural network can be trained to act as a surrogate for DFT computations. The advantage here is that running a forward pass through a neural network can be orders of magnitude faster than executing the corresponding DFT computation,^[^ ^30^, ^31^ ^]^ which enables screening over far larger compositional spaces. It is important to note that a neural network cannot be expected to produce results more accurate than the DFT data upon which it is trained. However, what it can offer is computational speed and strategic guidance for identifying scenarios that warrant more in‐depth analysis with more rigorous physics‐based models. A similar case can be made for the applicability of machine learning in molecular dynamics simulations.^[^ ^32^, ^33^ ^]^

Even when the procedure for a task is well‐understood, ML‐models can still offer value by enabling automation and faster workflows. Image recognition serves as a good example. While it's relatively straightforward for a human to take a photo of a reaction outcome and evaluate whether large crystals have formed, the task is monotonous and time‐consuming. A convolutional neural network can perform the same task but without a human in the loop,^[^ ^34^ ^]^ which is both cheaper and more time efficient, even if not necessarily more accurate. Another illustrative example is the automation of X‐ray diffraction (XRD) analysis for high‐throughput combinatorial experiments.^[^ ^35^, ^36^ ^]^

Yet another use case is clustering. When dealing with a large volume of unlabelled data, algorithms can be employed to group similar items together, revealing connections and patterns that might not be immediately apparent. These insights can serve as the foundation for subsequent studies aimed at developing more accurate physics‐based models.

### 2.2 Under the Hood: Data, Features, and Models

Machine learning models at the initial rung of the ML/AI‐ladder generally follow a similar workflow: data collection, data cleaning, feature selection, model selection, model training, and hyperparameter tuning using a subset of the data (i.e., the training and/or validation set), followed by model evaluation using the remaining data (i.e., the test set).^[^ ^37^, ^38^, ^39^ ^]^ What distinguishes the application of machine learning in the field of materials science from other domains is primarily the nature of the data collected and the specific features that are of importance.^[^ ^40^, ^41^, ^42^, ^43^, ^44^ ^]^

When discussing data in the realm of materials science, it is useful to differentiate among theoretical data, publicly available experimental data, and in‐house generated experimental data. Generally, datasets within the materials field tend to be relatively small, with a few notable exceptions. Among the exceptions are DFT databases like the Materials Project,^[^ ^45^ ^]^ NOMAD,^[^ ^46^ ^]^ Aflow,^[^ ^47^ ^]^ etc. which may have data for a few hundred thousand compounds.

These DFT databases are interesting not only because of their large size but also because they contain data on the materials’ crystal structure, from which much of the intrinsic properties of a material is derived. From an ML perspective, a current challenge is how to develop featurization schemes that effectively utilize the information contained within the DFT data. When working with truly large datasets, it may be possible to get away with using very simple features such as various one‐hot encoding schemes. One could for example imagine to only use atomic numbers as features. This is because more complex, expressive features can be learned during the training process, which is an approach commonly employed in for example image recognition.^[^ ^48^ ^]^ While DFT databases may be large in the context of materials science, they are still relatively small when compared to typical ML datasets. This necessitates the creation of richer, more informative features. Additionally, most ML models require feature vectors of consistent lengths for each material. Simpler featurization schemes are often based on the materials composition, with atomic features being averaged based on the stoichiometry of the compounds. Various versions of these exist,^[^ ^49^ ^]^ such as Magpie^[^ ^50^ ^]^ and Oliynk.^[^ ^51^ ^]^ While easy to compute, these featurisation schemes are position‐independent and thus overlook valuable structural data. By using the atomic coordinates, it is possible to construct more sophisticated and expressive features. Examples include sine matrices, aimed at generalizing the concept of molecular Coulomb matrices to periodic crystals;^[^ ^52^ ^]^ Smooth Overlap of Atomic Positions fingerprint (SOAP);^[^ ^53^ ^]^ Many‐Body Tensor Representations (MBTR);^[^ ^53^ ^]^ and Partial Radial Distribution Functions (PRDF).^[^ ^54^ ^]^ Yet another approach is the use of Graph Neural Networks (GNNs), which focus on the bonding information between atoms in the unit cell, rather than their atomic coordinates.^[^ ^55^, ^56^, ^57^ ^]^ Developing functional featurisation schemes for materials remains and open field of research, and there will be reasons to return to that topic in later papers.

Another valuable source of data comes from experimental results collected in large databases. Historically, the field of materials science has not excelled at creating open‐access experimental databases. A notable exception exists within the crystallographic community, which early on established standards for formatting, reporting, and storing crystallographic data. This proactive approach has led to the creation of databases such as the Crystallographic Open Database (COD)^[^ ^58^ ^]^ and the Cambridge Structural Database (CSD),^[^ ^59^ ^]^ each housing hundreds of thousands of crystal structures derived from diffraction measurements. These databases greatly complement the theoretical DFT databases discussed above.

Several factors contribute to the limited availability of experimental materials databases. First, experiments are not only challenging to execute but also costly and time‐consuming. Materials data is also highly heterogeneous, encompassing a wide array of synthesis and characterization techniques, each of which requires extensive metadata ontologies to make sense. Moreover, there are numerous different applications for materials, each emphasizing a distinct set of properties, which further complicates the data landscape. This has not been an environment that encourage strong cultures of open data sharing. Instead, the prevailing practice has been to visualize and describe selected data in academic papers without providing easy access to the raw data. Practises are, however, now gradually changing to the better. In part, this is a consequence of more researchers seeing the value in what is known as FAIR data treatment, i.e., that data should be made findable, accessible, interoperable, and reusable.^[^ ^60^, ^61^ ^]^ There are also an increasing number of funding agencies, governmental bodies, and publishers demanding data to be shared openly. In both cases, the popularisation of ML/AI‐modelling and the associated need for open data is catalyzing the process.

In addition to publicly available and proprietary databases, there is also in‐house data. Apart from the effort and resources involved in gathering new experimental data, and the limited amount of data that results in, it is often easier to work with. One advantage is internal consistency; it can be uniformly formatted from the start, the parameter space is well defined, missing values can be complemented, and data from failed experiments are accessible, which can significantly enhance model performance.^[^ ^59^ ^]^ Models derived from this type of data may be good for solving specific problems, but are typically narrow in scope, and often not very generalisable. Regarding model selection, a common practice is to explore a range of models available in frameworks like Scikit‐learn,^[^ ^14^ ^]^ or other frameworks that offer high‐level implementations of a wide array of traditional ML‐algorithms. There are plenty of excellent sources discussing the mathematics and implementation of such models in detail.^[^ ^18^, ^19^ ^]^

### 2.3 Consequences

Utilizing ML‐models in the way described in this section has the potential to accelerate research, uncover hidden patterns, and simplify the screening of new materials. At this stage, machine learning serves as a set of tools that, when properly implemented, is an indispensable part of modern research practises. Consequently, mastering these tools should be an essential part in any STEM‐education. However, while valuable, ML‐modelling at this level is not revolutionary in nature. It primarily involves employing robust statistical methods, translated into computer code, and adopting a mindset that treats all data – both positive and negative – as valuable assets. While perhaps not transformative, those who adopt machine learning techniques and this data‐centric mindset, are likely to experience increased productivity and can tackle more complex research questions.

## 3 The Second Rung: Robots, Automation, and Physical Manipulations

### 3.1 The Case for Automation

The next step up the complexity ladder occurs when machine learning models gain the ability to directly interface with physical laboratory equipment. At this stage, the models begin to use their predictive capabilities to autonomously manipulate the physical environment, by for example synthesizing new samples or generating new measurement data. The enabler for this direct interaction is robotics, which is intrinsically tied to the concept of automation.

Automation has since the industrial revolution served as a catalyst for enhancing efficiency, increasing throughput, reducing cost, and liberating humans from repetitive tasks. While academic research has not been immune to this trend, the complex and ever‐shifting nature of research activities has made them more challenging to automate compared to standardized industrial processes. Human dexterity and adaptability are hard to outcompete when it comes to moving samples around and manipulate vials, pipets, bottles, powders, and other things design for human operations. Consequently, automation in academic settings has largely been confined to specialized instruments capable of executing well‐defined, repetitive tasks, with sample exchangers and pipetting robots being prime examples. The investment cost, the skillset, and the commitment required for complete lab automation have also been limiting factors.

With cheaper hardware and better software, we are now witnessing a growing number of example of lab automation for high‐throughput synthesis and characterization platforms that can automate an increasing number of consecutive steps in the research process.^[^ ^36^, ^62^, ^63^, ^64^, ^65^, ^66^, ^67^ ^]^ Those systems are often referred to as Materials Acceleration Platforms (MAPs),^[^ ^64^, ^68^ ^]^ and can vary in complexity and in the number of tasks they can execute.

One type of MAPs is based on microfluidic systems. Those allow for precise mixing of small liquid volumes and high‐time resolution monitoring of reactions through optical methods.^[^ ^69^, ^70^, ^71^, ^72^, ^73^, ^74^, ^75^, ^76^, ^77^ ^]^ These systems offer the advantages of minimal sample volumes, high precision, and high throughput with potentially thousands of experiments per day. However, they are constrained in terms of the types of chemistries that can be investigated and the in‐line characterization techniques that can be applied.

A more versatile approach involves the use of pipetting robots or robotic arms for manipulation of vials and pipets and standard liquid‐based synthesis, as well as transfer of samples between various measurement stations.^[^ ^63^, ^78^ ^]^ This enables the exploration of a broader range of chemistries and allows for workflows that incorporate a variety of standard equipment. Essentially anything that fits on a lab bench could be integrated in such workflows. At the even higher end of the complexity spectrum are autonomous, self‐navigating collaborative robots that can be integrated into standard lab environments. These advanced robots are capable, in principle, of executing any manual task that a human researcher could perform.^[^ ^66^, ^79^ ^]^ Burges et al. have proved a nice example of such a system exploring new photocatalysts.^[^ ^64^ ^]^

When executed effectively, robot‐assisted lab automation can substantially increase sample throughput compared to traditional manual experimentation. Moreover, it enhances data consistency by minimizing human variability, and it simplifies automatic logging of data and related metadata. Robot‐assisted lab work is at its core not fundamentally different from traditional artisanal lab work. However, the sheer increase in data output made possible within given time and budget constraints can cause this quantitative advantage to morph into a qualitative change as well. A parallel of such a transformation can be seen for computer power where more powerful computers have not only accelerated computations but also unlocked entirely new possibilities. Lab automation may be transformative in the same way. If you can suddenly synthesize and characterize samples at a rate 1000 times faster than before, it opens the door to exploring entirely new research questions.

While lab automation offers significant advantages, it is not a one‐size‐fits‐all solution. High‐quality robotic systems targeting laboratory work remain costly and are relatively scarce. Moreover, the learning curve to fully utilize these systems can be steep. In a dynamic lab setting where research focus frequently shifts, the cost‐effectiveness of robotic automation may also be questionable for short‐term projects. However, the trajectory is promising. The cost of robotic solutions is gradually decreasing, while their availability, user‐friendliness, and adaptability are on the rise. As these trends continue, robot‐assisted experimentation is poised to become an increasingly appealing option for accelerating materials research.

Even though robotic automation offers several advantages, it is important to remember that in traditional setups, robots only execute tasks explicitly programmed by humans. Moreover, even with the most efficient robots, we can only explore a tiny fraction of the synthetic parameter space, except for the most constrained problems. Lab automation, therefore, does thus not remove the intellectual challenges inherent in experimental research. It is still up to the human researcher to formulate relevant questions, define the boundary of the parameter space to explore, decide which experiments that should be conducted, and to interpret the data generated.

### 3.2 Combining Robots with Machine Learning

**Figure 2:**
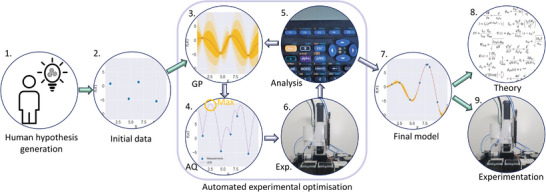
Workflow for Bayesian optimization combined with robotics for accelerated experimentation. It is up to the human researcher to formulate hypothesises and set the experimental boundaries 1). With initial data and insights 2) a Gaussian process (GP) can be used to generate a prior 3), here illustrated for 1D‐data. Based on the Gaussian process, an acquisition function (AQ) is computed and optimized 4) which guide a robot system 5) to do a new synthesis and set of measurements. That generated data is then automatically analyzed 6) after which the prior is updated (3). The process is then repeated until a stopping criterion is reached, A final model is then precented 7), which can be used as the basis for new models and theory 8), or more dedicated experiments 9).

Another step up in complexity involves integrating robotics and lab automation with machine learning and artificial intelligence. This has the potential to augment not just the manual but also the intellectual aspects of research. One emerging concept in this realm is closed‐loop experimentation, which aims to minimize human involvement in the research process as much as possible (**Figure** **2**).^[^ ^63^, ^64^ ^]^ The core idea behind this concept is the recognition that the development of new functional materials often resembles an optimization problem. Typical research objectives include identifying material compositions with specific properties, as well as determining the synthetic conditions that enable these materials to achieve the desired microstructure and how to incorporate them into devices. These challenges usually involve navigating large, nonlinear, multidimensional parameter spaces under the hypothesis that a specific region within these spaces will yield the desired results. Even with a relatively small number of variables and a coarse grid, conducting an exhaustive search becomes impractical within any reasonable budget. A critical task, therefore, is to wisely select experiments to minimize the path travelled toward the goal while navigating these large multidimensional parameter spaces.

Several strategies exist for automating optimization, with Bayesian optimization^[^ ^78^, ^80^, ^81^, ^82^, ^83^, ^84^ ^]^ being a popular example. Genetic algorithms is another example.^[^ ^85^ ^]^ A core idea behind Bayesian optimization is to initiate the process with a few randomly selected experiments, or to leverage prior experience, to construct a preliminary model of the system. This model is often termed the hypothesis function, or the prior. Gaussian processes are a popular choice for these functions as they provide not only interpolated estimates but also uncertainty estimates. The goal in designing the prior is to ensure it can be easily optimized to achieve the overarching research objective. This optimized prior then serves as a guide for selecting the next experiment to conduct. After executing the recommended experiment, the newly acquired data can be used to refine the existing prior model. By iteratively performing these steps, researchers can dramatically reduce the number of necessary experiments, enabling more efficient navigation through the parameter space compared to traditional design of experiments methods.^[^ ^86^, ^87^ ^]^

Traditional iterative experimental development often follows a similar logic, even if this process is not always formalized or consciously acknowledged. An automated and mathematically formalized approach eliminates human ambiguity and removes the bottleneck caused by manual data evaluation and experimental planning after each test. However, the application of robot‐assisted Bayesian optimization in materials science is still a rather new practice. Much development remains in terms of best practices, user‐friendliness, and cost‐effectiveness until closed‐loop systems become standard equipment. As robots become more affordable, successful case studies increase in number, and software integrations grows increasingly sophisticated, we can anticipate that these methods will eventually become standard practices in the academic research toolkit.

### 3.3 Consequences

Closed‐loop experimentation represents a qualitative leap forward in the ongoing quest to conduct more research with fewer resources. By combining the high‐throughput capabilities of automation with the efficiency of Bayesian optimization, which automates data analysis and guides subsequent experiments, substantial advantages can be realized. While this approach may not be applicable to every research problem, when it is effective, it has the potential to dramatically accelerate the pace of discovery.^[^ ^67^ ^]^ Compared to traditional methods of experimentation and conventional automation, this approach represents more than just an increase in throughput. It marks a significant qualitative shift by introducing autonomous decision‐making. Here we are not only replacing and/or expanding the human capacity for manual labor and number crunching. We are also augmenting intellectual aspects of the research process. This is particularly evident in how the system autonomously hypothesizes the best subsequent experiment after each round of measurements. We may today be at the initial stages of this development, but continued progress may from a laboratory perspective fundamentally change the relationship between humans and machines. The dynamic may shift from one where machines serve to augment human scientific capabilities to one where humans assist the machine to work as efficiently as possible. This transition will undoubtedly occur in incremental steps, but it is worth contemplating how these changes could reshape the skill sets required for competitive materials research.

The human experience of research may change at this level of artificial intelligence. Even so, it will not make humans obsolete. The closed‐loop experimentation paradigm can accelerate optimization processes, handle the practical aspects of experiments, and even automate intermediate data analysis and decision‐making. However, the intellectual underpinnings of the research – identifying what is worth to explore, formulating research questions, and decide what to optimize – will still rely on human visions and ingenuity. It will also be up to humans to set the boundaries for the optimization, and to interpret the significance of the results. The intellectual load put upon the human researcher could actually be expected to increase. While machines may handle an increasing share of the operational workload, there will be an increased demand for generating hypothesizes and formulating research questions. There will also be an increased demand for the strategic and interpretive aspects of research.

Operating within this new paradigm will require a certain skillset. Programming, tinkering with robot equipment, advanced data analysis, and strategic experimental planning are already valuable competencies, but they are likely to become even more important for researchers aiming to stay competitive. These skills should therefore be more heavily emphasised in research educations. The pace of hypothesis testing will also intensify. Gone are the days when a single good idea could fuel months of data collection and analysis in the lab. With automated systems, preliminary answers could arrive in a matter of days, or even hours, necessitating a continuous stream of new ideas for exploration. This quick turnover will place greater demands on researchers to generate hypotheses and adapt more rapidly to the results. The ability to think fast, broad, and innovatively will thus become even more valuable in the research landscape of the future.

Closed‐loop‐experimentation may also require roles that could be classified as less skilled, although essential for system functioning. In principle, everything could be automated with enough resources. However, a cost‐benefit analysis will probably often favor flexible humans with dexterous hands for tasks like supplying clean substrates, vials, and pipettes, preparing stock solutions, weighing dry chemicals, packing up new deliveries, and waste management.

Another significant shift that closed‐loop experimentation could catalyze is the transformation of the types of services that laboratories can provide. Currently, it is common to offer what can be called “analysis as a service,” where samples are sent to an external lab for specialized testing. In the future, we may instead see the rise of what can be called “optimization as a service.” In this evolved model, instead of sending a sample, clients would provide the lab with specific boundary conditions and objectives. The lab would then use automated systems to identify the optimal conditions within the provided parameter space given the stated objectives. This could dramatically expand the scope and efficiency of laboratory services.

## 4 The Third Rung: Generative Models and Hypothesis Generation

The next rung up the AI/ML ladder encompasses a broader development of artificial intelligence with potentially far‐reaching implications for numerous aspects of human life, including material science. At this level of sophistication we encounter large language models (LLMs) like GTP‐4,^[^ ^6^ ^]^ LaMDA,^[^ ^88^ ^]^ and LLaMA,^[^ ^89^ ^]^ that recently have attracted lots of attention for their ability to generate text with human qualities based on neural networks utilizing the transformer architecture.^[^ ^90^, ^91^ ^]^ This technology is still in its early stages and is evolving rapidly, and its future potential remains exciting but uncertain. Nevertheless, we can already today start to see how these models could be utilized in material science research.^[^ ^92^ ^]^

The core strength of large language models lies in text generation, making them particularly useful for writing applications.^[^ ^93^ ^]^ They already today excel at condensing complex text into more digestible formats, such as educational materials or public communication documents.^[^ ^94^, ^95^ ^]^ They have a tendency to be factual incorrect and they are not yet capable of writing scientific papers that would pass per‐review – we think. However, they are sufficiently good at simpler writing exercises to cause some panic in the educational sector, and when used as a writing assistant they could improve the text quality of most average writers.

If trained on comprehensive scientific literature databases those models have the capability to mine, scan, summarize, and analyse the academic literature.^[^ ^96^, ^97^, ^98^, ^99^ ^]^ While they are not designed to replace human experts with specialized domain knowledge, these models could significantly streamline and simplify the process of conducting literature reviews.^[^ ^98^, ^100^ ^]^ This could become an invaluable tool for researchers trying to understand a field and identify emerging trends and key discoveries within it.

One of the most intriguing possibilities, however, lies in the potential for these AI systems to generate new hypotheses based on existing knowledge, which can then be explored experimentally. Such generative AI could recommend novel material systems, suggest alterations to existing systems, or assist in brainstorming innovative methodologies, techniques, or experiments worth pursuing. At the time of writing, state‐of‐the‐art models, like Chat‐GPT, still find it challenging to produce hypotheses robust enough to serve as the foundation for a scholarly article. It is not impossible, but to succeed requires both domain knowledge and a bit of luck. However, it is not all that far from being there,^[^ ^101^, ^102^ ^]^ and it can quite consistently provide topics, questions, and ideas that could form a good basis for a Ph.D. research project.^[^ ^103^ ^]^ Given the rapid pace of advancements in this field, we can anticipate that these models will mature into highly effective tools for academic research.

This marks a significant step into what has traditionally been an exclusively human intellectual domain. However, in its current form, rather than posing a risk of replacing humans, this technology has the potential to significantly amplify our capacity for generating hypotheses. A plausible workflow might involve employing generative AI as an assistant to brainstorm a list of ten novel hypotheses, followed by utilizing its capabilities to sift through existing literature to assess the plausibility, originality, and significance of each hypothesis. Armed with this groundwork, the human researcher can then make an informed decision about which hypothesises that seems most promising and design new experiments accordingly.

One distinguishing feature of ML/AI‐models at this level of abstraction, compared to lower rungs on the AI ladder, is model size and generalizability. Earlier, we discussed smaller models that typically are trained on a moderate volume of data generated either in‐house or extracted from specialized databases. For these models, a basic understanding of the underlying mathematics and code implementation is essential to unlock their full potential. For generative models, like LLMs or text‐to‐image generation, the situation is different. These are instead expansive models trained on vast datasets – essentially, a substantial portion of the text or image data available on the internet. Consequently, what the average user interacts with is not the complex mathematical underpinnings, but the trained model and its user interface. While a considerable amount of effort will be invested in refining and evolving these models, the primary concern for material scientists will be how to harness the capabilities of these models and how to use them as building blocks in new workflows.

Large language models are not the only generative technologies that recently have attracted massive attention also outside academic circles. The field of text‐to‐image generation, exemplified by techniques such as stable diffusion, has also gained a lot of attention. To date, these technologies have primarily been employed for artistic endeavours by generating stunning images, which among other things have sparked conversations about the essence of art.^[^ ^104^, ^105^, ^106^ ^]^ While the potential applications within material science remain unclear at the moment, it is not unreasonable to anticipate that compelling use‐cases will eventually be found also for this technology.

### 4.1 Consequences

Sophisticated generative artificial intelligence is still a relatively recent development, and we are in the process of discovering all the ways it can augment, enhance, and accelerate our research efforts. The rapid advancements these technologies are currently experiencing add another a layer of uncertainty, making it challenging to foresee the full extent of their future capabilities. However, one thing is clear: these systems have the potential to become invaluable and transformative research tools. Those AI systems could streamline the process of identifying patterns and trends in scientific literature, enhance the quality of scientific writing and public dissemination, assist in coding for data analysis and visualization, and perhaps most crucially, amplify our ability to generate research hypotheses. For research groups aiming to maintain long‐term competitiveness, it would be highly advisable to closely monitor these technological advancements, and experiment with how they could be used to augment, improve, and extend the research process. At this stage of sophistication, researchers will not lose their work to an AI agent; but they may lose it to researchers that have figured out how to use AI‐systems to improve the quality and throughput of their own research.

## 5 The Fourth Rung: Orchestration and Autonomy

We are currently witnessing rapid growth in increasingly capable AI systems, each designed to handle specific tasks. The next step up the AI ladder is less about inventing new technologies and more about creatively combining already existing ML/AI modules. This can be conceptualized as orchestrating sub‐modules into larger, more versatile systems. For example, if a large language model is integrated with a voice‐to‐text module, a language translation module, domain‐specific dictionaries, a physics engine, a mathematics program, a web crawler, a CAD program, and a text‐to‐voice module, the creation of a comprehensive personal digital assistant becomes an achievable goal.

To envision a hypothetical use case, let's say a user speaks to the computer, asking if there is any substance that could be used as a dye with strong absorption in the green wavelength range, is soluble in toluene, non‐toxic, and is not too expensive. The voice‐to‐text module would first transcribe the spoken question into text. Then, the language model would interpret the meaning of the query. Specialized algorithms would mine scientific literature for potential candidates, while web crawlers would scrape commercial websites for pricing and availability data. Additional specialized modules, including a physics engine, could perform the necessary computations to evaluate the suitability of the target molecules. Finally, the text‐to‐voice module would present the user with a suggestion and inquire whether an order for the selected molecule should be placed.

Integrating various modules to operate cohesively is no easy task, but it may be a less daunting task than it was to develop all the specialized narrow AI models. Progress in this area is already underway, exemplified by initiatives from companies like Hugging Face which have showed that it is possible to use LLMs as a controller to manage existing AI models to solve sophisticated AI tasks in different modalities and domains.^[^ ^8^ ^]^ Examples in material science where LLMs are connected to robotic experimentation are still few but include recrystallization experiments,^[^ ^107^ ^]^ successful performance of catalysed cross‐coupling reactions,^[^ ^108^ ^]^ and synthesis of humidity colorimetric sensors.^[^ ^109^ ^]^ The reports currently available on this topic has the character of initial proof‐of‐concepts but provide an indication of where we may be heading.

A valuable distinction to make is between the orchestration of digital and physical systems. The integration of digital components is likely to precede their physical counterparts, primarily because it doesn't necessitate the development of new robotic hardware. The closed‐loop materials platforms mentioned earlier serve as an example of how digital models can be integrated with robotics. For the foreseeable future, robotics will likely remain the most significant challenge in these types of integrative efforts. However, in principle, there are no inherent limitations preventing us from expanding these systems to incorporate increasingly sophisticated and capable models, both in the realms of robotics and AI.

Robots are generally specialized to excel in a narrow set of tasks. For example, a pipetting robot is adept at pipetting but cannot do anything more. Even a robotic arm, while somewhat more flexible, has its own set of limitations. The problem for robots is the bar set by humans, which repertoire of motions is incredibly diverse and flexible and guided by complex sensory input and computational power – most of which we take for granted. Steve Wozniak's “coffee test” serves as a popular illustration of this challenge. A human can easily walk into an unfamiliar kitchen and make a cup of coffee, a feat that is extraordinarily difficult for a robot unless the kitchen has been specifically designed for robotic coffee preparation. This limitation is referred to as the problem of universal robotics. To fully automate a physical lab environment and eliminate the need for human intervention, significant advancements in universal robotics will be necessary.

### 5.1 Consequences

**Figure 3:**
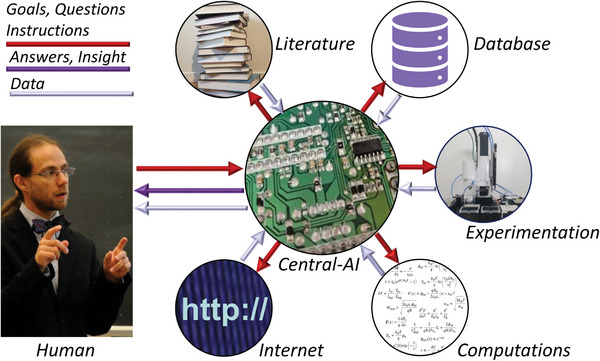
Illustration of the human AI interaction in an orchestrated system which has a central AI unit, using an LLM as an interface, which have access to the scientific literature, physics and mathematics engines, can search the internet, store data in databases, and control robot driven experimentation.

In a future with affordable universal robotics, we could envision these technologies to be fully integrated with the AI models previously discussed (**Figure** **3**). This would enable fully autonomous scientific facilities. At such facilities, all we would need to provide are ideas, hypotheses, objectives, and capital. In return, we would receive data, materials, and insights. This would significantly alter the role of human researchers. For instance, the hands‐on, practical skills that currently constitute a large part of many Ph.D. students' daily work would become a thing of the past. Instead, we would need a stronger emphasis on data science, a deeper understanding of the theoretical aspects of our chosen field, a more comprehensive view of the bigger picture, and a clear sense of what we seek to discover – and why we want it. Particularly, this last point may define our enduring role in the scientific process. As automation takes over many tasks that today are considered intellectual labor, what remains uniquely human is the ability to weave the broader narrative of “why.” Ultimately, the core purpose of research is to cater to human needs, aspirations, and curiosity. As long as we can articulate those objectives, there will be a place for humans in the research process, albeit different from what it is today.

## 6 The Fifth Rung and Beyond: Toward the Singularity

Even with the remarkable advancements covered in the preceding sections, there are still numerous rungs to ascend on the AI ladder toward ever‐greater complexity, sophistication, and capability. At the heart of this discussion is the concept of General Artificial Intelligence (AGI), which is an AI system that has reached the cognitive flexibility to perform any intellectual task that a human can do. Whether AGI is an unattainable goal or an imminent reality is a subject under lively debate.^[^ ^110^, ^111^ ^]^ However, one thing is certain: if AGI becomes a reality, it will open a Pandora's box of unknowns, with strong arguments suggesting it could be one of the most transformative developments in human history. The core argument posits that an AGI, unencumbered by the biological constraints that limit human intelligence, could initiate a positive feedback loop.^[^ ^112^ ^]^ In this loop, the AGI would continually use its computational prowess to refine and enhance its own algorithms, potentially leading to a state of superintelligence.^[^ ^113^ ^]^ Once this self‐amplifying cycle is established, the concept of a technological singularity – the hypothetical point in time at which technological growth becomes uncontrollable and irreversible – is not an implausible scenario.^[^ ^114^ ^]^ Under those conditions, the question we set out with in the beginning – “What role will material scientists play in the era of artificial intelligence?” – transitions from a subject suitable for educated speculation to one more appropriately confined to the realm of science fiction.

## 7 Concluding Remarks

We are currently witnessing rapid advancements in increasingly sophisticated machine learning and artificial intelligence systems. Even if general artificial intelligence may not be imminent, these technologies provide invaluable tools that can significantly accelerate efforts in material science in for example developing and discovering new functional materials aimed at addressing urgent global challenges. In this paper, we have arranged ML/AI‐approaches based on their level of sophistication, spanning from simple regression analysis to AI‐guided robotic systems, generative models for hypothesis generation, and the orchestration of specialized AI modules as a stepping‐stone toward general artificial intelligence. As these models increase in sophistication, so does their potentially transformative impact on material development. However, this also necessitates a shift in the skill sets required by researchers. We anticipate that skills that will increase in value will include data science, programming, a deep understanding of the theoretical aspects of the chosen field, and a clear vision of what we aim to discover – and why it is important to do so.

Looking forward, it is unlikely that the typical materials researcher will be replaced by AI agents within the next few decades. However, they may find themselves outperformed by researchers who have successfully harnessed the power of AI to enhance both the quality and efficiency of their work. Therefore, the overarching advice for those wishing to stay competitive is to invest in understanding and mastering the emerging ML/AI methods and models, and to experiment in how one can leveraging their capabilities to improve both the quantity and quality of research.

## Conflict of Interest

The authors declare no conflict of interest.

## Use of AI

Maybe somewhat ironically given that this is a perspective about the usefulness of AI, the intellectual work in this perspective is completely made by humans. However, ChatGPT 4 has been used as a writing assistance to suggest improvements of the grammar and the flow of the text, which is a great use for non‐native English speakers.
